# Recent Advances in the Discovery and Biosynthetic Study of Eukaryotic RiPP Natural Products

**DOI:** 10.3390/molecules24081541

**Published:** 2019-04-18

**Authors:** Shangwen Luo, Shi-Hui Dong

**Affiliations:** State Key Laboratory of Applied Organic Chemistry, College of Chemistry and Chemical Engineering, Lanzhou University, Lanzhou 730000, China; luosw@lzu.edu.cn

**Keywords:** natural product, RiPP, ribosomally synthesized, post-translationally modified peptides

## Abstract

Natural products have played indispensable roles in drug development and biomedical research. Ribosomally synthesized and post-translationally modified peptides (RiPPs) are a group of fast-expanding natural products attribute to genome mining efforts in recent years. Most RiPP natural products were discovered from bacteria, yet many eukaryotic cyclic peptides turned out to be of RiPP origin. This review article presents recent advances in the discovery of eukaryotic RiPP natural products, the elucidation of their biosynthetic pathways, and the molecular basis for their biosynthetic enzyme catalysis.

## 1. Introduction

### 1.1. Common Features of RiPP Biosynthesis

**Figure 1:**
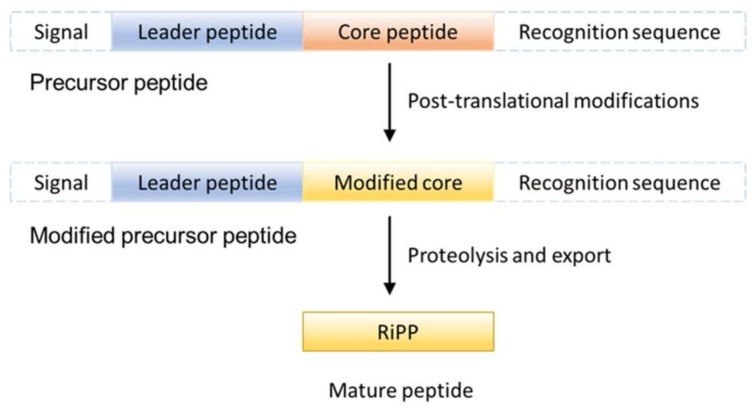
General Ribosomally synthesized and post-translationally modified peptide (RiPP) natural products biosynthetic pathway. Adapted from Reference [1].

Ribosomally synthesized and post-translationally modified peptides (RiPPs) are ribosomally synthesized and post-translationally modified peptide natural products. As their name indicates, all RiPP natural products are encoded by structural genes and are initially synthesized as precursor peptides by ribosome (Figure 1). In most RiPPs, the precursor peptide consists of a sequence-conserved amino N-terminal leader peptide and a hypervariable core sequence. Many eukaryotic precursor peptides, as described in this review, have a carboxyl C-terminal recognition sequence that is important for excision and cyclization [1]. In general, the precursor peptide is first synthesized by the ribosome. Then, the core peptide is subjected to post-translational modifications, many of which are guided by leader peptides and recognition sequences. Finally, the leader sequences and recognition sequences are removed by proteolysis to generate mature peptides. Notably, some post-translational modifications are leader/recognition sequence independent, catalyzed after removal of the flanking sequences. 

Due to the fact that RiPP core peptides are directly translated from open reading frames (ORFs) in the genomes of the producing organisms, genome mining algorithms and toolkits were developed to correlate the mature RiPP with its corresponding biosynthetic gene cluster (BGC) and to use that information to search for more homologous BGCs. On the other hand, by analyzing the sequence of a putative homologous BGC, the sequence and even the raw structure of its corresponding mature RiPP can be predicted as well [2,3].

### 1.2. Designation of RiPP Families

Nisin, produced by *Lactococcus lactis*, is one of the longest known RiPPs first reported in the 1920s and has been used as a food preservative since the 1960s [1]. Its structure is characterized by the presence of lanthionine residues, giving the name lanthipeptides (for lanthionine-containing peptides) to this family of RiPPs. It was not until recently that the biosynthetic mechanisms for lanthionine residues in nisin were revealed [4,5,6]. The family of lanthipeptides are further divided into four classes according to different domain organizations of the key biosynthetic enzymes that install the thioether crosslinks in their characteristic lanthionine residues [1].

Historically, RiPP families have been either defined based on the producing organisms, such as microcins from Gram-negative bacteria, or their bioactivities, such as bacteriocins that exhibit antibacterial activities. A consensus was reached in 2013 within the scientific community to designate RiPPs based on their structural and biosynthetic commonality [1]. Accordingly, a variety of RiPP families were defined, such as linaridins, proteusins, linear azol(in)e-containing peptides (LAPs), cyanobactins, thiopeptides, bottromycins, lasso peptides, microviridins, and sactipeptides.

### 1.3. Engineering Potential of RiPP Antibiotics

Many RiPPs natively display potent antibiotic activities, such as lasso peptides, lanthipeptides, and thiopeptides. Biosynthetic studies of those antibiotic RiPPs showed that their biosynthetic pathways are modular. More importantly, many of RiPP biosynthetic enzymes are promiscuous and can tolerate alternative substrates. This plasticity in RiPP biosynthesis gives rise to engineering efforts in making new-to-nature compounds with higher potency and better bioavailability. As more RiPP biosynthetic pathways are revealed and more RiPP biosynthetic enzymes are thoroughly investigated, the therapeutic potential of RiPPs will be significantly increased by those engineering efforts [7].

Bacterial RiPPs have been more extensively studied than eukaryotic RiPPs in the past. However, eukaryotic RiPPs are equally important in providing novel chemical scaffolds and valuable enzymatic transformations. To this end, this review article briefly introduces characteristics of various RiPP natural products from eukaryotic organisms, including the discovery of novel eukaryotic RiPPs and their structural characteristics, emphasizes on recent advances in eukaryotic RiPP biosynthetic studies, and, finally, discusses the unique features of eukaryotic RiPP biosynthesis comparing to bacterial RiPP biosynthesis.

## 2. Fungal RiPP

### 2.1. RiPP from Basidiomycetes

#### 2.1.1. Amatoxins and Phallotoxins

**Figure 2:**
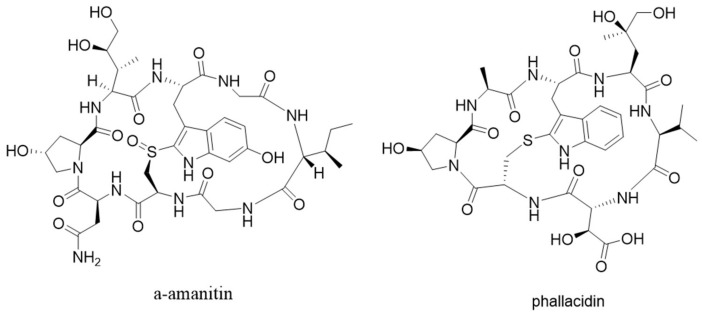
Structures of α-amanitin and phallacidin.

Mushrooms in the genus *Amanita* account for most of fatal mushroom poisonings [8]. Their toxicity is caused by a group of bicyclic peptides named amatoxins. Amatoxins are also biosynthesized by mushrooms in other unrelated genera, such as *Galerina*, *Lepiota*, and *Conocybe*. They can cause liver failure and death by inhibiting ribonucleic acid (RNA) polymerase II [9]. Amanita mushrooms are also responsible for producing a structurally related group of toxins, called phallotoxins, which are orally inactive but toxic when injected. Phallotoxins act by stabilizing F-actin, and have been utilized to stain the cytoskeleton (Figure 2) [9].

**Figure 3:**
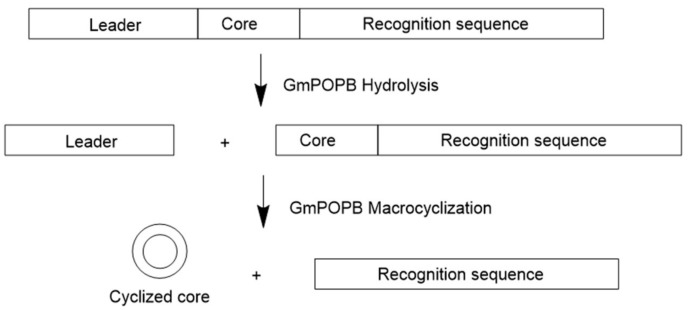
Schematic of prolyl oligopeptidase (POPB) catalyzed proteolysis and cyclization. GmPOPB is the POPB from *Galerina marginata* species. Adapted from Reference [13].

Genome survey sequencing revealed that amatoxins and phallotoxins are biosynthesized via ribosomal pathways [8,10]. It was also shown that genes encoding precursor peptides for amatoxins and phallotoxins are prevalent in toxic mushrooms and form a large family, known as the MSDIN family for the first five conserved amino acid residues in the precursor peptides [11,12]. Members of the MSDIN family are characterized by a hypervariable core region flanked by conserved leader and recognition sequences. Moreover, the core is flanked by invariant proline residues that act as proteolytic targets by a prolyl oligopeptidase (POP), named POPB (Figure 3).

**Figure 4:**
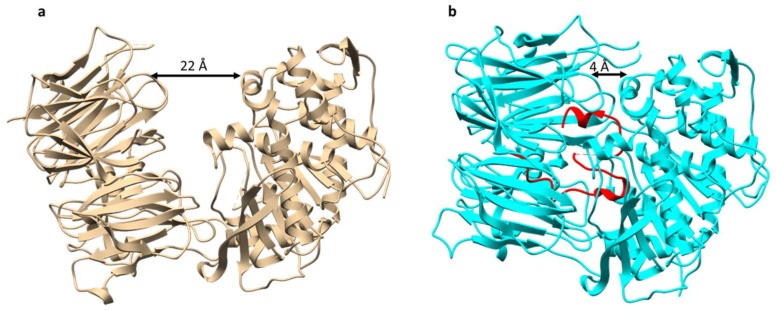
Overall structures of prolyl oligopeptidase POPB. (**a**) Structure of POPB from *Galerina marginata* species unbound to substrate (apoGmPOPB) in tan (PDB 5N4F), (**b**) S577A mutant of GmPOPB bound to 35-mer peptide (PDB 5N4C), S577A mutant in cyan, 35-mer peptide in red.

POPB is a member of the POP family of serine proteases. It differs from conventional POP (such as POPA that is also present in *Amanita* mushrooms) in that it catalyzes two nonprocessive reactions: Hydrolysis of leader peptide following the proline residue, and transpeptidation to form macrocycle of the core peptide [13]. This two-step mechanism of POPB catalysis was also supported by kinetic and structural studies (Figure 4). The enzyme first hydrolyzes N-terminal leader by the removal of 10 residues from a 35-residue precursor. The resulting 25 amino-acid peptide is conformationally trapped and forced to be released. After dissociation from the enzyme, the 25-mer is conformationally rearranged and rebounded by the enzyme. This process is possibly directed by the C-terminal follower peptide. Finally, the follower peptide is removed and the core peptide is macrocyclized in the active site of the same enzyme [14]. Due to its unusual two-step mechanism, high substrate tolerance in the core region, and satisfying kinetic efficiency, POPB has been exploited as a general catalyst for peptide macrocyclization [15].

#### 2.1.2. Borosins

**Figure 5:**
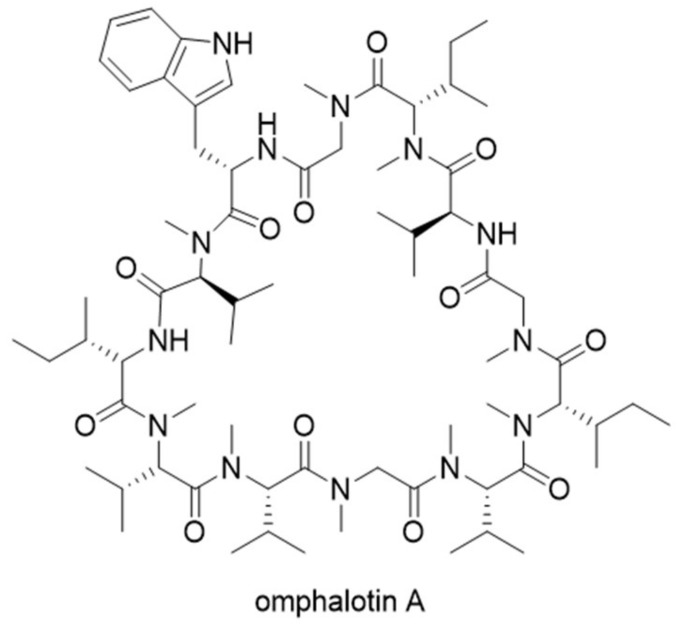
Structure of omphalotin A.

Omphalotin A was isolated from the basidiomycete *Omphalotus olearius* with potent and selective nematotoxic activity. The structure of omphalotin A is characterized by a peptidic macrocycle with nine *N*-methylations on the amide backbone (Figure 5). It was postulated that omphalotin A was biosynthesized by a nonribosomal peptide synthetase (NRPS) pathway, because backbone *N*-methylation had never been observed for RiPP pathways. It was not until 2017 that a RiPP biosynthetic gene cluster, *oph*, was confirmed to be responsible for producing omphalotin A by two groups in parallel [16,17]. Even more surprisingly, the precursor peptide of omphalotin A is not present as a stand-alone substrate for post-translational modifications, but rather fused to the C-terminal of a protein with sequence homology to *S*-adenosylmethionine (SAM)-dependent methyltransferases [18,19]. The gene encoding this fusion protein was named *ophA*. Additional experiments showed that OphA autocatalytically methylates its own C terminus in a sequential manner from N to C terminus, followed by cleavage and cyclization by the prolyl oligopeptidase OphP to form omphalotin [16]. Van der Velden et al. [17] proposed the name “borosins” after the ancient mythological symbol Ouroboros for this new family of RiPPs.

**Figure 6:**
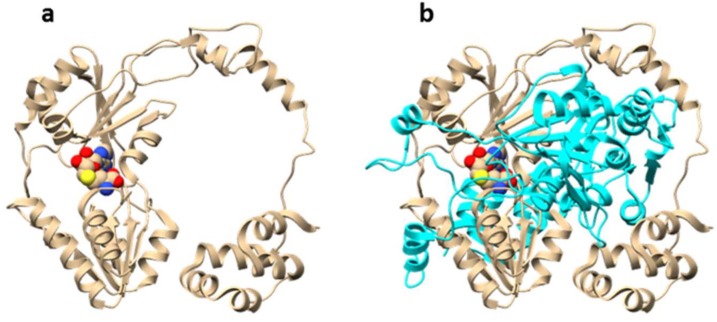
Overall structure of dbOphMA (PDB 6MJF). dbOphMA is OphA homolog from *Dendrothele bispora* species. (**a**) dbOphMA monomer bound to *S*-adenosyl homocystein (SAH), (**b**) interlocking organization of dbOphMA dimer.

The molecular mechanism of OphA automethylation was proposed based on structural studies by two groups in parallel (Figure 6) [20,21]. OphA acts by forming a homodimer, with each monomer resembling the appearance of a ring. In the co-complex structure, the C-terminal core peptide in monomer A sits into the methyltransferase active-site of monomer B (and vice versa), giving the dimer the appearance of two interlocked rings [20]. This structural arrangement results in substrate proximity and suggests an acid-base catalysis mechanism. The amide nitrogen is first deprotonated with the help of a basic amino acid residue (possibly arginine). The resulting negative charge is stabilized by tyrosine residues in close proximity. Finally, the proximity of a reactive SAM molecule promotes the alkylation reaction [21].

### 2.2. RiPP from Ascomycetes

#### 2.2.1. Dikaritins

##### Ustiloxins

**Figure 7:**
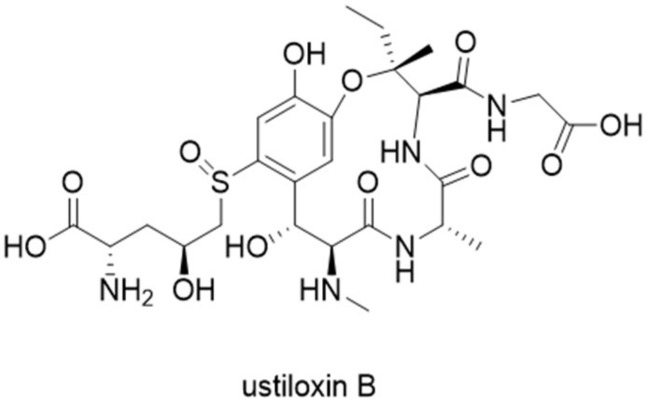
Structure of ustiloxin B.

Ustiloxins are the first example of natural products that are biosynthesized by RiPP pathways in filamentous fungi. The study of ustiloxin biosynthetic pathway represents the first example of complete RiPP gene cluster characterization in fungi [22]. Ustiloxin B was originally discovered from plant pathogenic fungus *Ustilaginoidea virens* with phytotoxic activity by inhibiting microtubule assembly [23]. The structure of ustiloxin B consist of a Tyr-Ala-Ile-Gly (YAIG) tetrapeptide and contains unusual norvaline modification on the hydroxylated tyrosine residue (Figure 7). The biosynthetic origin of ustiloxin B remained unknown until the genome mining method MIDDAS-M was developed for the detection of natural product biosynthetic gene clusters in fungi [24,25]. MIDDAS-M is the abbreviation of motif-independent de novo detection algorithm for secondary metabolite biosynthetic gene clusters. By scoring transcription levels of all putative gene clusters in *Aspergillus flavus* under different culture conditions, the MIDDAS-M method identified the gene cluster for ustiloxin B, named the *ust* cluster, which was later confirmed by knockout studies.

**Figure 8:**
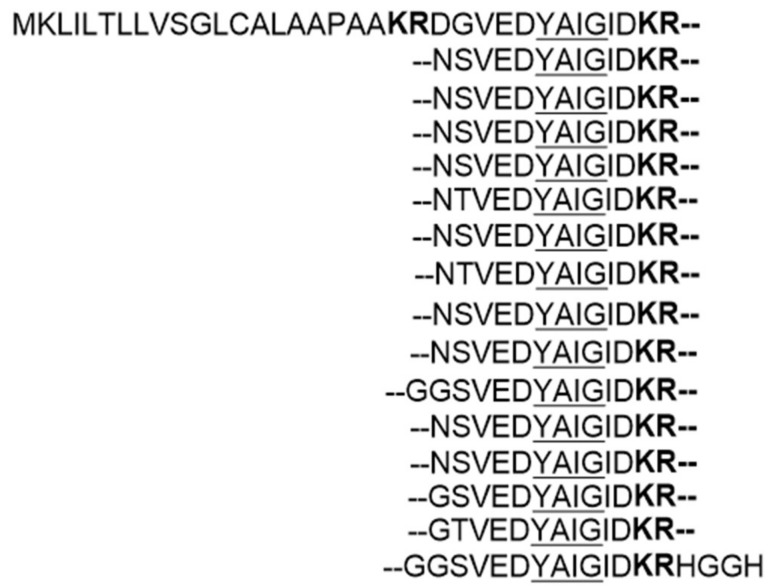
Sequence of UstA from *Aspergillus flavus*. KR motifs to be recognized by Kex2 protease are marked in bold. Repeated YAIG core sequences are underlined. Adapted from Reference [22].

In depth sequence analysis of the cluster revealed that the precursor peptide UstA contains 16-fold repeats of the YAIG core, and that each repeated core is flanked by conserved ED and KR motifs which are likely necessary for recognition by post-translationally modifying enzymes (Figure 8). Moreover, the N-terminal of UstA is a signal peptide-like sequence that also contains a KR motif. The overall organization of UstA, and the fact that KR motif is a known recognition site for Kex2 protease, which is a type of universal serine proteases, strongly suggest that ustiloxin B is a RiPP and that *ustA* encodes the precursor peptide [22]. Accordingly, the biosynthetic gene cluster of ustiloxin B in its original host, *U. virens*, was also characterized and confirmed to be a RiPP cluster [26].

**Figure 9:**
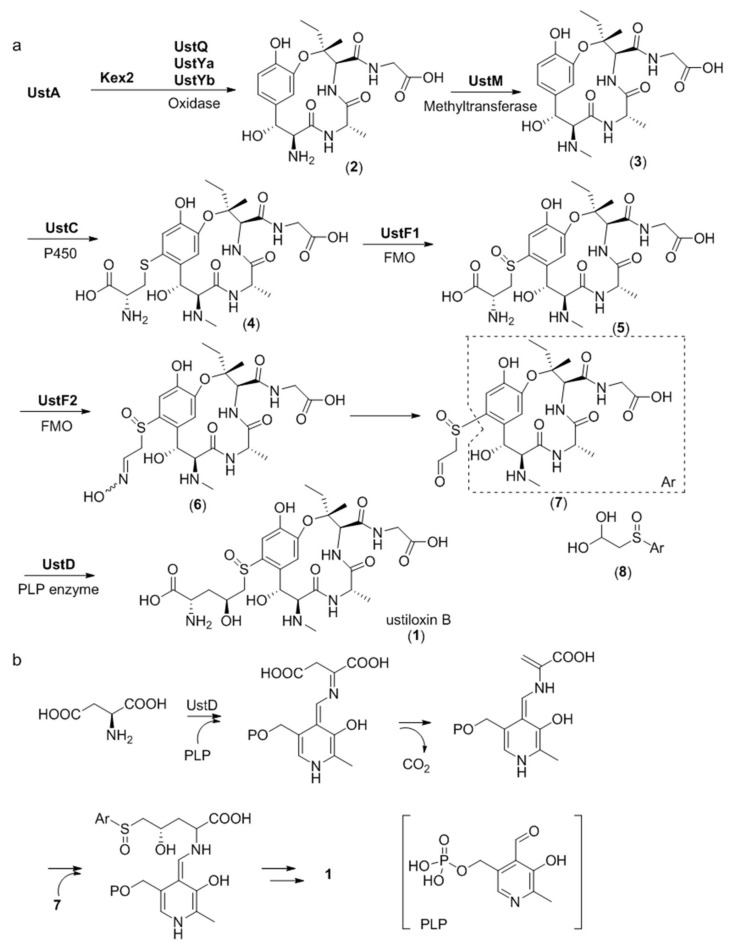
Proposed biosynthetic pathway for ustiloxin B. (**a**) Overall biosynthetic scheme, (**b**) Proposed mechanism of UstD catalyzed reaction. P450 = cytochrome P450 monooxygenase, FMO = flavoprotein monooxygenase, PLP = pyridoxal 5′-phosphate. Adapted from Reference [27].

The entire biosynthetic pathway of ustiloxin B has been studied in detail by gene inactivation, heterologous expression, and in vitro biosynthetic enzyme functional reconstitution (Figure 9a) [27]. Gene disruption studies revealed that the three genes *ustQYaYb* are essential to give the first intermediate **2**. UstQ is a tyrosinase homolog. UstYa/UstYb are mutual homologs containing the DUF3328 motif and have no homology with functionally known enzymes. Heterologous expression of *ustQYaYb* in *Aspergillus oryzae* gave **2** as the sole product. Based on these results, it was speculated that the UstA precursor is first digested into 16 trideca-/tetradecapeptides by Kex2 proteases before cyclization by UstQYaYb. However, the proteases that removes the N- and C- terminal sequences flanking the core are still unknown. UstM is a methyl transferase. Introduction of *ustM* into *ustQYaYb* transformants generated **3**. UstF1/UstF2 are Class B bifunctional flavoprotein monooxygenases (FMO). Purified maltose binding protein (MBP)-tagged UstF1/UstF2 showed yellow color and strong absorption at 450 nm, indicating binding of flavin adenine dinucleotide (FAD). Incubating **4** with UstF1 in the presence of nicotinamide adenine dinucleotide phosphate (NADPH) resulted in **5**, which was transformed into an entgegen/zusammen (*E/Z*) mixture of **6** after incubating with UstF2 in the presence of NADPH. Treatment of **6** with 0.1% trifluoroacetic acid (TFA) afforded **8**, a hydrate form of **7**. *ustD* gene showed homology with pyridoxal 5’-phosphate (PLP)-dependent enzyme. Incubating **8** with MBP-tagged UstD in the presence of PLP and aspartic acid generated ustiloxin B. The reaction mechanism of UstD was studied by incubating the enzyme with PLP and aspartic acid and treating the reaction mixture with dansyl chloride. The above experiment generated dansylated alanine, indicating that UstD catalyzes decarboxylation of aspartate to form an enamine, which acts as a nucleophile and reacts with **7** to give **1** (Figure 9b). Given that *ustYa/ustYb* are located near the precursor peptide gene *ustA*, combined queries of homologs of *ustYa/ustYb* and *ustA* identified 94 homologous clusters in *Aspergilli* genome sequences.

##### Asperipins

**Figure 10:**
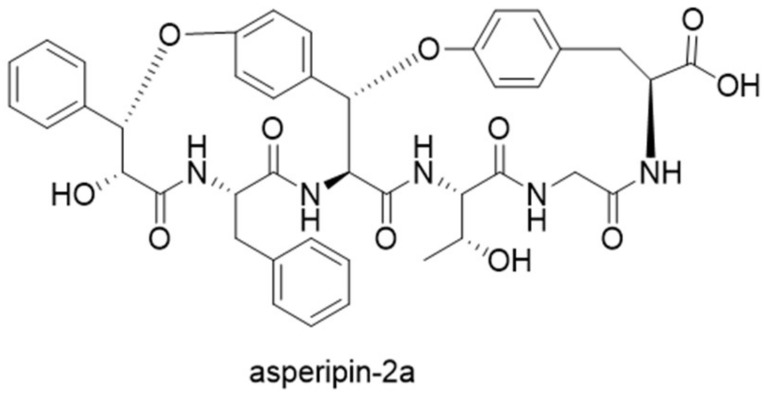
Structure of asperipin-2a.

Guided by the finding of 94 precursor peptide gene candidates by querying *ustYa/ustYb* and *ustA* in combination, a new cyclic peptide asperipin-2a was isolated from *Aspergillus flavus* [28]. Although asperipin-2a has high homology to ustiloxins in their gene clusters, they have distinct structural characteristics. Asperipin-2a has a hexa-peptidic core sequence of FYYTGY, forming a bicyclic structure connected by ether linkages between tyrosine side chains and β-carbons (Figure 10). The putative biosynthetic gene cluster for asperipin-2a is only composed of four genes: A precursor peptide gene *aprA*, a *ustYa/ustYb* homolog *aprY*, a transporter *aprT*, and an isoflavone reductase *aprR*. Heterologous expression of asperipin-2a gene cluster in *Aspergillus oryzae* showed that *aprY* is essential for biosynthesizing asperipin-2a, and indicated a sequential oxidative macrocyclization function for AprY [29].

##### Phomopsins

**Figure 11:**
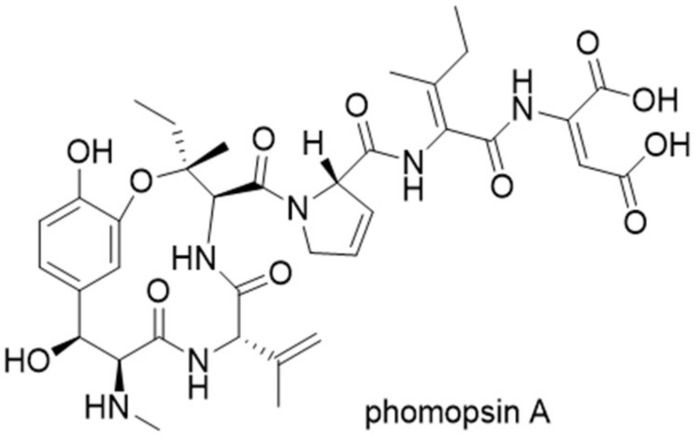
Structure of phomopsin A.

Phomopsins are a group of cyclic hexapeptide produced by the plant pathogenic fungus *Phomopsis leptostromiformis*. The structures of phomopsins are characterized by a 13-member macrocyclic ring formed by ether linkage between tyrosine and isoleucine (Figure 11). They are potent antimitotic compounds that target the vinca domain of tubulin, causing liver disease in livestock fed on infected plants [30]. A RiPP gene cluster was confirmed to be responsible for producing phomopsins by analyzing the genome sequence of *P. leptostromiformis* ATCC 26115 [31]. Similar to ustiloxin B precursor peptide gene *ustA*, *phomA*, the precursor peptide gene for phomopsins is also arranged in the same pattern. The N-terminal of PhomA is a signal-peptide like leader sequence, followed by eight repeats of core peptide flanked by conserved KR motifs. Knockout studies showed that the tyrosinase PhomQ is essential for phomopsin biosynthesis and is likely involved in forming the cyclic scaffold. In vitro enzymatic assays revealed that the methyltransferase PhomM installs methyl groups onto the N-terminal α-amino group. A search for PhomA, PhomQ, and PhomM homologous proteins in the National Center for Biotechnology Information (NCBI) database resulted in the identification of 27 similar gene clusters, suggesting the presence of a family of fungal RiPP natural products. Because these compounds appear to associate with strains of the subkingdom Dikarya, the name “dikaritins” was proposed for this new family of peptides. A global sequence similarity network was constructed for all of the putative proteins from the identified gene clusters, showing that these gene clusters contain a set of highly conserved proteins, including PhomA homologs, PhomQ homologs, PhomR-like zinc finger transcription-regulating proteins, and S41 family peptidases. Noteworthy is the presence of DUF3328 proteins in all of the gene clusters, such as UstYa/UstYb and AprY, whose role in dikaritin biosynthesis remains to be elucidated [31].

#### 2.2.2. Epichloёcyclins

Epichloёcyclins were discovered from grass endophytic fungi belonging to the genus *Epichloё*. MS/MS analyses indicated their structure characteristics to be a hepta-peptidic ring formed by oxidative cyclization on the tyrosine residue, and methylations on the lysine residue. Detailed structures of epichloёcyclins remain to be determined. The precursor peptide gene for epichloёcyclins was identified from fungal transcripts in endophyte-infected grasses and designated *gigA* (grass induced gene). GigA is composed of a signal sequence at its N-terminal, followed by four repeats of sequences containing the core peptide and conserved motifs, such as KR recognition site for Kex2 protease. Epichloёcyclins are the first example of RiPP natural products found in mutualistic symbiotic fungus, suggesting a possible bioactive role [32]. Whether epichloёcyclins belongs to the family of dikaritins or forms its own family of RiPP remains to be determined until the full biosynthetic gene cluster of epichloёcyclins can be identified.

## 3. Plant RiPP

### 3.1. Cyclotides

**Figure 12:**
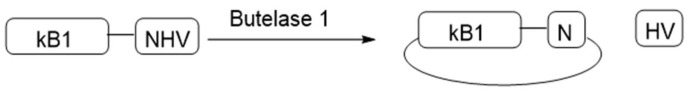
Schematic of butelase 1 catalyzed cyclization. kB1 containing 29 amino acid residues is the core peptide of plant cyclotide kalata B1. Asp-His-Val (NHV) is the C-terminal sequence recognized by butelase 1. Adapted from Reference [39].

**Figure 13:**
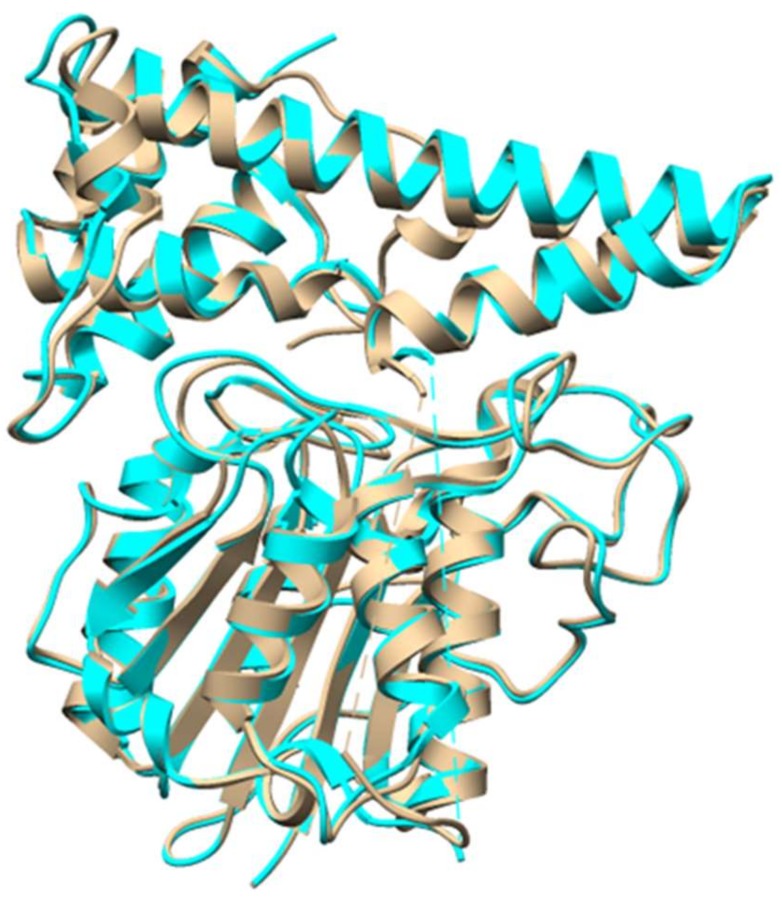
Crystal structure of butelase 1 (tan, PDB 6DHI) overlaid with structure of conventional AEP from *Oldenlandia affinis* (cyan, PDB 5H0I).

Cyclotides are plant derived RiPPs that are characterized by a head-to-tail cyclic peptide backbone and a signature cyclic cystine knot (CCK) motif [33]. They were discovered from plants of the Rubiaceae, Violaceae, Cucurbitaceae, and Fabaceae families [34,35,36,37,38]. Due to their insecticidal activities, cyclotides were thought to be plant defense agents. The broad range of other biological activities, such as antiviral, antimicrobial, and cytotoxic activities made cyclotides attractive for pharmaceutical applications [1]. The precursors of cyclotides can be present as dedicated proteins, similar to other RiPPs from bacteria and fungi. However, it was found that cyclotide precursors in *Clitoria ternatea* (Fabaceae family) are embedded within an albumin precursor, indicating RiPPs might be much more common than has been thought [36,37]. Cyclotide precursors consists of an endoplasmic reticulum (ER) domain, a pro-region (PRO), an N-terminal region (NTR), and one or more copies of the core sequence. The protease that removes the leader remains to be characterized. Butelase 1, a Asx-specific peptide ligase from cyclotide producing *C. ternatea*, was characterized to be responsible for cyclotide backbone cyclization [39]. Butelase 1 has high sequence homology with asparaginyl endopeptidase (AEP), and indeed showed AEP activity. However, it recognizes the C-terminal Asn/Asp-His-Val (D/NHV) sequence and is capable of cyclizing various peptides of plant and animal origin with high catalytic efficiencies (Figure 12) [39]. A recent structural study revealed that the active site of butelase 1 has only subtle differences from conventional AEPs, suggesting its efficient macrocyclization activity may be attributed to its peptide binding region (Figure 13) [40]. A co-crystal structure of butelase 1 with its peptide substrate will help us understand the mechanism of macrocyclization. Due to its high promiscuity and fast kinetics, butelase 1 has been applied in protein labelling [41,42], chemoenzymatic synthesis of bacteriocins [43], generating cyclic peptides with non-native amino acids [44], decorating *E. coli* cell surfaces [45], making peptide dendrimers [46], and preparing C-to-C fusion proteins [47].

### 3.2. Orbitides

**Figure 14:**
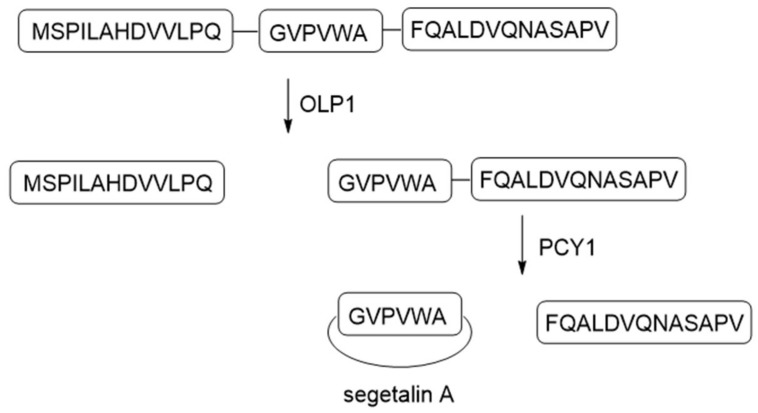
Schematic of segetalin A biosynthetic pathway. Letters in the figure represents single letter notations for amino acids. OLP1 is a serine protease. PCY1 is a peptide cyclase. Adapted from Reference [49].

**Figure 15:**
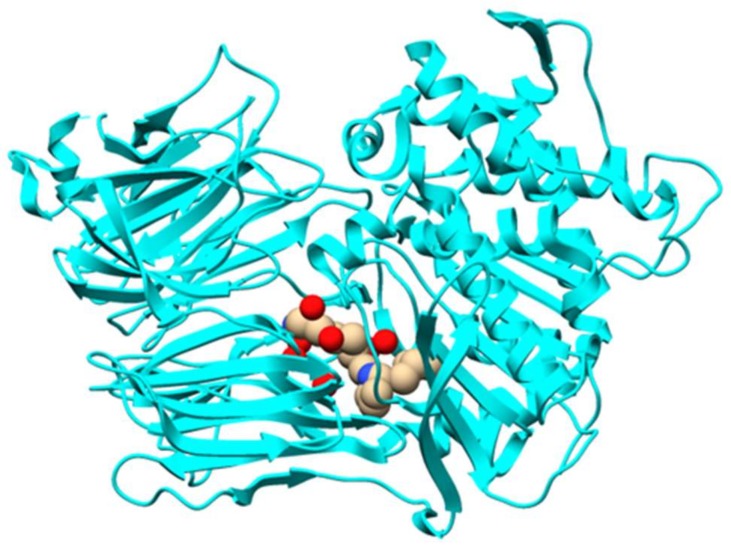
Overall structure of peptide cyclase PCY1 with follower peptide (PDB 5UW3).

Orbitides refer to N-to-C cyclized plant peptides that do not contain disulfides. They are produced by at least nine plant families: Annonaceae, Caryophyllaceae, Euphorbiaceae, Lamiaceae, Linaceae, Phytolaccaceae, Rutaceae, Schizandraceae, and Verbenaceae [1]. Similar to cyclotides, orbitide precursor peptides also contain multiple copies of core sequences, resulting in a single precursor to be processed to multiple cyclic peptides. The biosynthetic pathway of orbitide segetalin A has been studied in detail (Figure 14). The 32-amino acid precursor presegetalin A1 is first processed by the serine protease OLP1 to remove the N-terminal 15 residues. Then, the peptide cyclase PCY1 cleaves the C-terminal 13 amino acids, with concomitant macrocyclization of the remaining six residues to form segetalin A [48]. PCY1 is identified as a member of the S9A protease family that includes POP enzymes. Kinetic analysis showed that PCY1 has similar *k*_cat_ values, and five~10-fold higher *K*_M_ values comparing to butelase 1 involved in cyclotide macrocyclization. Crystal structures of PCY1 revealed its transamidation and cyclization mechanisms (Figure 15). Upon binding of the follower peptide, PCY1 is maintained in a closed state that precludes solvent from the active site, potentially limiting the competing hydrolysis reaction. A key residue His659 sits on a mobile loop, which contributes to two roles: Activating Ser nucleophile to form acyl-enzyme intermediate, and deprotonating the α-amine of the substrate for transamidation [49]. Using the obtained knowledge of PCY1 reaction molecular basis, a three residue C-terminal extension (F/I-Q-A/T) was designed to replace the native long recognition tail FQALDVQNASAPV, permitting PCY1 to work on synthetic substrates [50].

## 4. Animal RiPP

Marine snails, such as cone snails, are known to produce a variety of ribosomally synthesized and post-translationally modified peptide venoms. Those venomes are produced by predatory cone snails, injected into the prey, and lead to paralysis. The best characterized marine snail peptides are conopeptides, also known as conotoxins. It was estimated that more than 500 species of predatory marine *Conus* snails are capable of producing conotoxins [51,52]. Those *Conus* species can produce as many as 70,000 structurally diverse conotoxins [53,54]. Many conotoxins act by targeting ion channels, thus have been widely used as basic research tools in neuroscience. A number of conotoxins showed therapeutic potential due to their unparalleled potency and selectivity against a wide range of receptors and ion channels [55]. For example, Ziconotide, a calcium channel agonist isolated from *Conus magus*, was approved by the United States Food and Drug Administration (US FDA) in 2004 for the treatment of chronic pain. A detailed review of conopeptides discovery and biosynthesis was made in 2013, and more structures of conopeptides have been characterized since then [1,56,57,58].

The precursor peptide sequences of conotoxins were studied by analyzing the transcriptomes of cone snails. Those studies revealed that conotoxin precursor transcript sequences consist of three regions: An ER signal peptide, a mature peptide region, and pre-/postpropeptide regions [59,60]. The ER signal peptide sequence is highly conserved, whereas the mature peptide region is highly diverse [60]. Types of conotoxin post-translational modifications include disulfide-bond formation, proline hydroxylation, *O*-glycosylation on serine or threonine residues, and glutamate *γ*-carboxylation [61,62]. A web-based ConoServer database (conoserver.org) was established to record known structures of conopeptides, classifications, post-translational modifications, and their general statistics. Due to that conopeptide biosynthetic genes are not organized in clusters, biosynthetic studies of animal RiPPs are extremely challenging. The details of conotoxin biosynthetic pathways and the mechanism and molecular basis for their post-translational modifications still remain largely unexplored [55].

## 5. Discussion

Eukaryotic RiPP pathways have some special features comparing to bacterial RiPP pathways. As described above, many fungal and plant RiPPs have N-terminal recognition sequences in their precursor peptides. Moreover, C-terminal signal sequences are also common in eukaryotic RiPP precursors. For example, in the case of cyclotides (Section 3.1), an ER signal sequence is present in their precursor peptides [63]. In addition, the core region of eukaryotic precursor peptides often has several repeats of the core sequence, flanked by conserved motifs. Although this manner is also found in cyanobactin biosynthesis, whose cores are present as repetitive cassettes, it is not common in other bacterial RiPP pathways [64]. Even more surprisingly, some eukaryotic RiPP precursors are not encoded as stand-alone genes, but rather as fusion or chimeric proteins. For example, omphalotin A precursor is fused to the C-terminal of a post-translationally modifying enzyme methyltransferase (Section 2.1.2), and some cyclotide precursors are embedded within an albumin precursor (Section 3.1). The presence of precursor peptides fused to other structural genes underscores the possibility that eukaryotic RiPP natural products are much more common than have been found. Interestingly, the biosynthesis of many eukaryotic RiPPs involves an N-C macrocyclization catalyzed by proteases. For example, amatoxins are macrocyclized by POPB enzymes, cyclotides are formed by a head-to-tail cyclization catalyzed by butelase 1, and the N-to-C cyclization of orbitides are catalyzed by PCY1 proteases [65]. The resulting macrocyclic peptides are more resistant to protease degradation in physiological environments, thus having more potential to be developed into novel therapeutics.

RiPP natural products are promising candidates for developing novel therapeutics, as many RiPPs have shown significant biological activities and great engineering potential [7]. The studies of eukaryotic RiPP biosynthesis are relatively more challenging than their bacterial counterparts, mainly due to the more complex genomic context. This challenge can be compromised by the development of optimal computational tools for mining eukaryotic genomes [2,66,67]. As more RiPP natural products are discovered, and more RiPP biosynthetic pathways are revealed, this group of fast expanding natural products will continue to provide compounds for industrial applications, and to inspire engineering efforts on enzymatic machineries.

## References

[1] Arnison P.G., Bibb M.J., Bierbaum G., Bowers A.A., Bugni T.S., Bulaj G., Camarero J.A., Campopiano D.J., Challis G.L., Clardy J. (2013). Ribosomally synthesized and post-translationally modified peptide natural products: overview and recommendations for a universal nomenclature. Nat. Prod. Rep..

[2] Blin K., Wolf T., Chevrette M.G., Lu X.W., Schwalen C.J., Kautsar S.A., Duran H.G.S., Santos E.L.C.D.L., Kim H.U., Nave M., Dickschat J.S. (2017). antiSMASH 4.0-improvements in chemistry prediction and gene cluster boundary identification. Nucleic Acids Res..

[3] Tietz J.I., Schwalen C.J., Patel P.S., Maxson T., Blair P.M., Tai H.C., Zakai U.I., Mitchell D.A. (2017). A new genome-mining tool redefines the lasso peptide biosynthetic landscape. Nat. Chem. Biol..

[4] Li B., Yu J.P.J., Brunzelle J.S., Moll G.N., van der Donk W.A., Nair S.K. (2006). Structure and mechanism of the lantibiotic cyclase involved in nisin biosynthesis. Science.

[5] Ortega M.A., Hao Y., Zhang Q., Walker M.C., van der Donk W.A., Nair S.K. (2015). Structure and mechanism of the tRNA-dependent lantibiotic dehydratase NisB. Nature.

[6] Ortega M.A., Hao Y., Walker M.C., Donadio S., Sosio M., Nair S.K., van der Donk W.A. (2016). Structure and tRNA Specificity of MibB, a Lantibiotic Dehydratase from Actinobacteria Involved in NAI-107 Biosynthesis. Cell Chem. Biol..

[7] Hudson G.A., Mitchell D.A. (2018). RiPP antibiotics: biosynthesis and engineering potential. Curr. Opin. Microbiol..

[8] Hallen H.E., Luo H., Scott-Craig J.S., Walton J.D. (2007). Gene family encoding the major toxins of lethal Amanita mushrooms. Proc. Natl. Acad. Sci. USA.

[9] Walton J.D., Hallen-Adams H.E., Luo H. (2010). Ribosomal Biosynthesis of the Cyclic Peptide Toxins of Amanita Mushrooms. Biopolymers.

[10] Luo H., Len-Adams H.E.H., Scott-Craig J.S., Walton J.D. (2012). Ribosomal biosynthesis of alpha-amanitin in Galerina marginata. Fungal Genet. Biol..

[11] Pulman J.A., Childs K.L., Sgambelluri R.M., Walton J.D. (2016). Expansion and diversification of the MSDIN family of cyclic peptide genes in the poisonous agarics Amanita phalloides and A-bisporigera. BMC Genom..

[12] Luo H., Cai Q., Luli Y., Li X., Sinha R., Hallen-Adams H.E., Yang Z.L. (2018). The MSDIN family in amanitin-producing mushrooms and evolution of the prolyl oligopeptidase genes. IMA Fungus.

[13] Luo H., Hong S.Y., Sgambelluri R.M., Angelos E., Li X., Walton J.D. (2014). Peptide Macrocyclization Catalyzed by a Prolyl Oligopeptidase Involved in alpha-Amanitin Biosynthesis. Chem. Biol..

[14] Czekster C.M., Ludewig H., McMahon S.A., Naismith J.H. (2017). Characterization of a dual function macrocyclase enables design and use of efficient macrocyclization substrates. Nat. Commun..

[15] Sgambelluri R.M., Smith M.O., Walton J.D. (2018). Versatility of Prolyl Oligopeptidase B in Peptide Macrocyclization. Acs Synth. Biol..

[16] Ramm S., Krawczyk B., Muhlenweg A., Poch A., Mosker E., Sussmuth R.D. (2017). A Self-Sacrificing N-Methyltransferase Is the Precursor of the Fungal Natural Product Omphalotin. Angew. Chem. Int. Edit..

[17] Van der Velden N.S., Kalin N., Helf M.J., Piel J., Freeman M.F., Kunzler M. (2017). Autocatalytic backbone N-methylation in a family of ribosomal peptide natural products (vol 13, pg 833, 2017). Nat. Chem. Biol..

[18] Bowers A.A. (2017). Methylating mushrooms. Nat. Chem. Biol..

[19] Aldemir H., Gulder T.A.M. (2017). Expanding the Structural Space of Ribosomal Peptides: Autocatalytic N-Methylation in Omphalotin Biosynthesis. Angew. Chem. Int. Edit..

[20] Song H., van der Velden N.S., Shiran S.L., Bleiziffer P., Zach C., Sieber R., Imani A.S., Krausbeck F., Aebi M., Freeman M.F. (2018). A molecular mechanism for the enzymatic methylation of nitrogen atoms within peptide bonds. Sci Adv..

[21] Ongpipattanakul C., Nair S.K. (2018). Molecular Basis for Autocatalytic Backbone N-Methylation in RiPP Natural Product Biosynthesis. Acs Chem. Biol..

[22] Umemura M., Nagano N., Koike H., Kawano J., Ishii T., Miyamura Y., Kikuchi M., Tamano K., Yu J.J., Shin-ya K. (2014). Characterization of the biosynthetic gene cluster for the ribosomally synthesized cyclic peptide ustiloxin B in Aspergillus flavus. Fungal Genet. Biol..

[23] Koiso Y., Li Y., Iwasaki S., Hanaoka K., Kobayashi T., Sonoda R., Fujita Y., Yaegashi H., Sato Z. (1994). Ustiloxins, Antimitotic Cyclic-Peptides from False Smut Balls on Rice Panicles Caused by Ustilaginoidea Virens. J. Antibiot..

[24] Umemura M., Koike H., Nagano N., Ishii T., Kawano J., Yamane N., Kozone I., Horimoto K., Shin-ya K., Asai K. (2013). MIDDAS-M: Motif-Independent De Novo Detection of Secondary Metabolite Gene Clusters through the Integration of Genome Sequencing and Transcriptome Data. PloS ONE.

[25] Umemura M., Koike H., Machida M. (2015). Motif-independent de novo detection of secondary metabolite gene clusters - toward identification from filamentous fungi. Front. Microbiol..

[26] Tsukui T., Nagano N., Umemura M., Kumagai T., Terai G., Machida M., Asai K. (2015). Ustiloxins, fungal cyclic peptides, are ribosomally synthesized in Ustilaginoidea virens. Bioinformatics.

[27] Ye Y., Minami A., Igarashi Y., Izumikawa M., Umemura M., Nagano N., Machida M., Kawahara T., Shin-ya K., Gomi K. (2016). Unveiling the Biosynthetic Pathway of the Ribosomally Synthesized and Post-translationally Modified Peptide Ustiloxin B in Filamentous Fungi. Angew. Chem. Int. Edit..

[28] Nagano N., Umemura M., Izumikawa M., Kawano J., Ishii T., Kikuchi M., Tomii K., Kumagai T., Yoshimi A., Machida M. (2016). Class of cyclic ribosomal peptide synthetic genes in filamentous fungi. Fungal Genet. Biol..

[29] Ye Y., Ozaki T., Umemura M., Liu C.W., Minami A., Oikawa H. (2019). Heterologous production of asperipin-2a: proposal for sequential oxidative macrocyclization by a fungi-specific DUF3328 oxidase. Org. Biomol. Chem..

[30] Cormier A., Marchand M., Ravelli R.B.G., Knossow M., Gigant B. (2008). Structural insight into the inhibition of tubulin by vinca domain peptide ligands. EMBO Rep..

[31] Ding W., Liu W.Q., Jia Y.L., Li Y.Z., van der Donk W.A., Zhang Q. (2016). Biosynthetic investigation of phomopsins reveals a widespread pathway for ribosomal natural products in Ascomycetes. Proc. Natl. Acad. Sci. USA.

[32] Johnson R.D., Lane G.A., Koulman A., Cao M.S., Fraser K., Fleetwood D.J., Voisey C.R., Dyer J.M., Pratt J., Christensen M. (2015). A novel family of cyclic oligopeptides derived from ribosomal peptide synthesis of an in planta-induced gene, gigA, in Epichloe endophytes of grasses. Fungal Genet. Biol..

[33] Craik D.J., Daly N.L., Bond T., Waine C. (1999). Plant cyclotides: A unique family of cyclic and knotted proteins that defines the cyclic cystine knot structural motif. J. Mol. Biol..

[34] Saether O., Craik D.J., Campbell I.D., Sletten K., Juul J., Norman D.G. (1995). Elucidation of the Primary and 3-Dimensional Structure of the Uterotonic Polypeptide Kalata B1. Biochemistry.

[35] Ireland D.C., Colgravel M.L., Nguyencong P., Daly N.L., Craik D.J. (2006). Discovery and characterization of a linear cyclotide from Viola odorata: Implications for the processing of circular proteins. J. Mol. Biol..

[36] Giang K.T.N., Zhang S., Ngan T.K.N., Phuong Q.T.N., Chiu M.S., Hardjojo A., Tam J.P. (2011). Discovery and Characterization of Novel Cyclotides Originated from Chimeric Precursors Consisting of Albumin-1 Chain a and Cyclotide Domains in the Fabaceae Family. J. Biol. Chem..

[37] Poth A.G., Colgrave M.L., Lyons R.E., Daly N.L., Craik D.J. (2011). Discovery of an unusual biosynthetic origin for circular proteins in legumes. Proc. Natl. Acad. Sci. USA.

[38] Giang K.T.N., Lian Y.L., Pang E.W.H., Phuong Q.T.N., Tran T.D., Tam J.P. (2013). Discovery of Linear Cyclotides in Monocot Plant Panicum laxum of Poaceae Family Provides New Insights into Evolution and Distribution of Cyclotides in Plants. J. Biol. Chem..

[39] Nguyen G.K.T., Wang S.J., Qiu Y.B., Hemu X., Lian Y.L., Tam J.P. (2014). Butelase 1 is an Asx-specific ligase enabling peptide macrocyclization and synthesis. Nat. Chem. Biol..

[40] James A.M., Haywood J., Leroux J., Ignasiak K., Elliott A.G., Schmidberger J.W., Fisher M.F., Nonis S.G., Fenske R., Bond C.S. (2019). The macrocyclizing protease butelase 1 remains auto-catalytic and reveals the structural basis for ligase activity. Plant J..

[41] Cao Y., Nguyen G.K.T., Tam J.P., Liu C.F. (2015). Butelase-mediated synthesis of protein thioesters and its application for tandem chemoenzymatic ligation. Chem. Commun..

[42] Nguyen G.K.T., Cao Y., Wang W., Liu C.F., Tam J.P. (2015). Site-Specific N-Terminal Labeling of Peptides and Proteins using Butelase 1 and Thiodepsipeptide. Angew. Chem. Int. Edit..

[43] Hemu X., Qiu Y.B., Nguyen G.K.T., Tam J.P. (2016). Total Synthesis of Circular Bacteriocins by Butelase 1. J. Am. Chem. Soc..

[44] Nguyen G.K.T., Hemu X., Quek J.P., Tam J.P. (2016). Butelase-Mediated Macrocyclization of d-Amino-Acid-Containing Peptides. Angew. Chem. Int. Edit..

[45] Bi X.B., Yin J., Nguyen G.K.T., Rao C., Halim N.B.A., Hemu X., Tam J.P., Liu C.F. (2017). Enzymatic Engineering of Live Bacterial Cell Surfaces Using Butelase 1. Angew. Chem. Int. Edit..

[46] Cao Y., Nguyen G.K.T., Chuah S., Tam J.P., Liu C.F. (2016). Butelase-Mediated Ligation as an Efficient Bioconjugation Method for the Synthesis of Peptide Dendrimers. Bioconjugate Chem..

[47] Harmand T.J., Bousbaine D., Chan A., Zhang X.H., Liu D.R., Tam J.P., Ploegh H.L. (2018). One-Pot Dual Labeling of IgG 1 and Preparation of C-to-C Fusion Proteins Through a Combination of Sortase A and Butelase 1. Bioconjugate Chem..

[48] Barber C.J.S., Pujara P.T., Reed D.W., Chiwocha S., Zhang H.X., Covello P.S. (2013). The Two-step Biosynthesis of Cyclic Peptides from Linear Precursors in a Member of the Plant Family Caryophyllaceae Involves Cyclization by a Serine Protease-like Enzyme. J. Biol. Chem..

[49] Chekan J.R., Estrada P., Covello P.S., Nair S.K. (2017). Characterization of the macrocyclase involved in the biosynthesis of RiPP cyclic peptides in plants. Proc. Natl. Acad. Sci. USA.

[50] Ludewig H., Czekster C.M., Oueis E., Munday E.S., Arshad M., Synowsky S.A., Bent A.F., Naismith J.H. (2018). Characterization of the Fast and Promiscuous Macrocyclase from Plant PCY1 Enables the Use of Simple Substrates. ACS Chem. Biol..

[51] Olivera B.M., Gray W.R., Zeikus R., Mcintosh J.M., Varga J., Rivier J., Desantos V., Cruz L.J. (1985). Peptide Neurotoxins from Fish-Hunting Cone Snails. Science.

[52] Olivera B.M., Rivier J., Clark C., Ramilo C.A., Corpuz G.P., Abogadie F.C., Mena E.E., Woodward S.R., Hillyard D.R., Cruz L.J. (1990). Diversity of Conus Neuropeptides. Science.

[53] Livett B.G., Gayler K.R., Khalil Z. (2004). Drugs from the sea: Conopeptides as potential therapeutics. Curr. Med. Chem..

[54] Davis J., Jones A., Lewis R.J. (2009). Remarkable inter- and intra-species complexity of conotoxins revealed by LC/MS. Peptides.

[55] Akondi K.B., Muttenthaler M., Dutertre S., Kaas Q., Craik D.J., Lewis R.J., Alewood P.F. (2014). Discovery, Synthesis, and Structure Activity Relationships of Conotoxins. Chem. Rev..

[56] Lebbe E.K.M., Ghequire M.G.K., Peigneur S., Mille B.G., Devi P., Ravichandran S., Waelkens E., D’Souza L., De Mot R., Tytgat J. (2016). Novel Conopeptides of Largely Unexplored Indo Pacific Conus sp.. Mar. Drugs.

[57] Reimers C., Lee C.H., Kalbacher H., Tian Y., Hung C.H., Schmidt A., Prokop L., Kauferstein S., Mebs D., Chen C.C. (2017). Identification of a cono-RFamide from the venom of Conus textile that targets ASIC3 and enhances muscle pain. Proc. Natl. Acad. Sci. USA.

[58] Turner A.H., Craik D.J., Kaas Q., Schroeder C.I. (2018). Bioactive Compounds Isolated from Neglected Predatory Marine Gastropods. Mar. Drugs.

[59] Woodward S.R., Cruz L.J., Olivera B.M., Hillyard D.R. (1990). Constant and Hypervariable Regions in Conotoxin Propeptides. EMBO J..

[60] Moller C., Melaun C., Castillo C., Diaz M.E., Renzelman C.M., Estrada O., Kuch U., Lokey S., Mari F. (2010). Functional Hypervariability and Gene Diversity of Cardioactive Neuropeptides. J. Biol. Chem..

[61] Bandyopadhyay P.K., Colledge C.J., Walker C.S., Zhou L.M., Hillyard D.R., Olivera B.M. (1998). Conantokin-G precursor and its role in gamma-carboxylation by a vitamin K-dependent carboxylase from a Conus snail (vol 273, pg 5447, 1998). J. Biol. Chem..

[62] Bandyopadhyay P.K., Garrett J.E., Shetty R.P., Keate T., Walker C.S., Olivera B.M. (2002). gamma-glutamyl carboxylation: An extracellular posttranslational modification that antedates the divergence of molluscs, arthropods, and chordates. Proc. Natl. Acad. Sci. USA.

[63] Shafee T., Harris K., Anderson M., Craik D.J. (2015). Chapter Eight - Biosynthesis of Cyclotides. Advances in Botanical Research.

[64] Gu W., Dong S.-H., Sarkar S., Nair S.K., Schmidt E.W., Moore B.S. (2018). Chapter Four - The Biochemistry and Structural Biology of Cyanobactin Pathways: Enabling Combinatorial Biosynthesis. Methods in Enzymology.

[65] Ongpipattanakul C., Nair S.K. (2018). Biosynthetic Proteases That Catalyze the Macrocyclization of Ribosomally Synthesized Linear Peptides. Biochemistry.

[66] Medema M.H., Fischbach M.A. (2015). Computational approaches to natural product discovery. Nat. Chem. Biol..

[67] Van der Lee T.A.J., Medema M.H. (2016). Computational strategies for genome-based natural product discovery and engineering in fungi. Fungal Genet. Biol..

